# Characterization of genomic DNA of lactic acid bacteria for activation of plasmacytoid dendritic cells

**DOI:** 10.1186/s12866-019-1458-y

**Published:** 2019-05-06

**Authors:** Akira Horie, Yasuyuki Tomita, Konomi Ohshio, Daisuke Fujiwara, Toshio Fujii

**Affiliations:** 1 Central Laboratories for Key Technologies, Kirin Co., Ltd., Japan 1-13-5, Fukuura Kanazawa Yokohama Kanagawa, Yokohama, 2360004 Japan; 2 Research Laboratories for Beverage Technologies, Kirin Co., Ltd., Yokohama, Japan

**Keywords:** Plasmacytoid dendritic cells, Type I interferon, *Lactococcus lactis* subsp. *lactis* LC-plasma, CpG, TLR9, G + C contents

## Abstract

**Background:**

*Lactococcus lactis* strain Plasma (LC-Plasma) possesses strong stimulatory activity for plasmacytoid dendritic cells (pDCs) via the TLR9-Myd88 pathway. To reveal the effective lactic acid bacteria (LAB) genome structure for pDCs stimulatory activity, we performed in vitro screening, using randomly selected 200 bp DNA fragments from the LC-Plasma genome.

**Results:**

We found that the CpG motif copy number in the fragments was positively and significantly correlated with pDCs stimulatory activity (*R* = 0.491, *p* < 0.01). However, the determination coefficient (*R*^*2*^*)* was 0.24, which means other factors affecte activity. We found that the G + C contents of the fragment showed a significant negative correlation with activity (*R* = − 0.474, *p* < 0.01). The correlation between pDCs stimulatory activity and the copy number of CpG motifs was greatly increased when DNA fragments were stratified by G + C contents.

We also performed bioinformatics analysis and a screening of LAB strains with high pDCs stimulatory activity. Species with a high copy number of CpG motifs in the low-G + C region of their genomes had higher probability of inducing high-pDCs stimulatory activity. *L. lactis* subsp. *lactis*, *Leuconostoc mesenteroides*, and *Pediococcus pentosaceus* were three typical examples of LAB that had high pDCs stimulatory activity.

**Conclusions:**

Our data suggested that the G + C content of DNA is one of the critical factors for pDCs stimulatory activity by DNA fragments. Furthermore, we found that the copy number in the low-G + C regions strongly affected the pDCs stimulatory activity of whole cells of LAB strains. These results should be useful for the design of new DNA fragments containing CpG motifs. This study also demonstrated an in silico screening method for identifying bacterial species that are able to activate pDCs.

**Electronic supplementary material:**

The online version of this article (10.1186/s12866-019-1458-y) contains supplementary material, which is available to authorized users.

## Background

The immunomodulatory effects of lactic acid bacteria (LAB) have attracted considerable attention over recent decades. Numerous animal studies and clinical studies have demonstrated that LAB have potent anti-allergy [1] and antiviral activity [2, 3]. Probiotic cell products that are responsible for immunomodulation are largely unknown but may involve some of the molecules that bind to the specific receptors of host cells, such as Toll-like receptors (TLRs). These include lipoteichoic acids (LTA), lipopolysaccharides (LPS), cell surface proteins, RNA, and DNA. Interestingly, several studies have suggested that strength of immunomodulatory activities depends on the species and strains of LAB [4–6].

Plasmacytoid dendritic cells (pDCs), a subset of dendritic cells (DCs), are immune cells that have a crucial function in immunological defense against viral infections [7, 8]. The pDCs originate in the bone marrow from myeloid and lymphoid precursors and require fms-like tyrosine kinase 3 ligand (Flt3L) for development. The pDCs sense DNA and RNA viruses through toll-like receptor 9 (TLR9) and TLR7, respectively, with subsequent production of interferon-alpha (IFN-α) [9]. This cytokine induces the expression of genes coding for anti-viral proteins such as MxA (myxovirus resistance A), viperin, and 2′-5′-oligoadenylate synthase. Several recent studies have revealed that pathogenic bacteria such as *Staphylococcus aureus* [10–12], *Neisseria meningitidis*, *Haemophilus influenzae* [12], and *Streptococcus pyogenes* [13] are able to enhance IFN-α production in both mice and humans. However, well-known probiotic LAB strains belonging to genera *Lactobacillus* and *Bifidobacterium* have not yet been reported to activate pDCs.

We previously found that a specific strain of LAB, LC-Plasma (synonym of *Lactococcus lactis* subsp. *lactis* JCM 5805) was able to stimulate production of IFN-α from murine pDCs [5]. Oral administration of LC-Plasma was found to result in significant immunomodulatory activity and profoundly enhanced antiviral activity in both mice and humans [14–17]. We also found that LC-plasma could stimulate pDCs via the TLR9-Myd 88 pathway [5]. The level of stimulation observed via the TLR2, TLR4, or TLR7-Myd 88 pathway was quite low. This suggested that CpG motifs from genomic DNA were the main Microbe Associated Molecular Patterns (MAMPs) for pDCs stimulation by LC-Plasma.

Unmethylated CpG motifs from bacterial genomes are ligands of TLR9 [18, 19]. Initially, 5′-GACGTC-3′, 5′-AGCGCT-3′, and 5′-AACGTT-3′ were identified as efficient immunostimulatory oligonucleotide (ISS-ODN) [20] and subsequent studies demonstrated that CpG-containing hexamers, CpG motifs, are able to stimulate B cells [18] and pDCs [21, 22]. Various types of CpG-motifs have been demonstrated as potent immunostimulatory DNA sequences [23]. Studies of ODNs with phosphorothioate backbones for clinical application revealed the key structure of ISS-ODNs. For example, Hartmann et al. studied the effect of base changes inside and outside of hexamers on activation of B and NK cells [24]. Lenert et al. studied the extended sequence preferences of both ISS-ODN and immuno-inhibitory ODN (INH-ODN) on B cells [25]. It has been proposed that 5′-RRCGYY-3′ and 5′-GTCGTT-3′ are optimal consensus sequences for B cell activation in mice and primates, respectively [18, 24]. The ISS-ODN-containing CpG motif for pDCs activation has only been identified very recently. The structural preference for ODN to activate pDCs is distinctly different from the ODN preference for B cells. 5′-RRCGRYCGYY-3′, 5′-RYCGYRTCGYR-3′, and 5′-RYCGRY-3′ have been shown to be the most efficient at activating pDCs [22].

In addition, several reports have suggested that more specific CpG motifs or even non-CpG sequences of LAB are critical for stimulation of B cell activity, including BL07 motifs in *Bifidobacterium longum* BB536 [26], OL-LB7 motifs in *Lactobacillus delbrueckii* [27], ID35 motifs in *Lactobacillus rhamnosus* GG [28], and OL-LG10 motif from *Lactobacillus gasseri* JCM 1131 [29].

In this study, we constructed a library of genomic DNA fragments from LC-Plasma and investigated the pDCs stimulatory activity of each fragment to identify the essential characteristic required for pDCs activation. As we expected, the CpG motif was necessary for active DNA fragments. However, we found that the total copy number of CpG motifs in each DNA fragment was not strongly correlated with its pDCs stimulatory activity and that the G + C content of genomic DNA fragments had a significant effect on the potential for pDCs activation. We also performed an in silico analysis of the copy number of CpG motifs in the genome LAB and found that the low G + C content of the genome has significant impact on pDCs stimulation.

## Results

CpG motifs are necessary for pDCs stimulatory activity of DNA fragments from LC-Plasma.

**Fig. 1:**
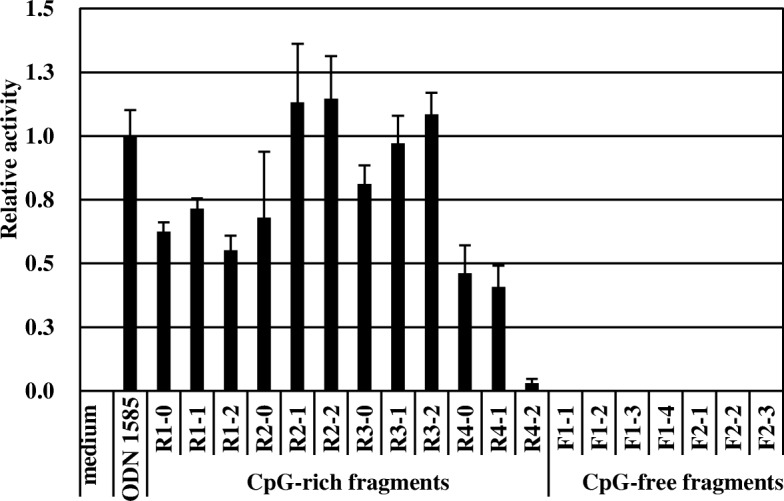
IFN-α induction by CpG-rich DNA fragments from *L. lactis* LC-Plasma. Flt3L-induced BM-DCs were stimulated by CpG-rich (R) or non-CpG (N) DNA fragments amplified from LC-Plasma genomic DNA. Each sample was added to cells at a final DNA concentration of 2 μg/mL Each value is the mean concentration ± S.D. for triplicate cultures

In order to confirm that the necessity of CpG motifs for pDCs stimulatory activity, we performed in vitro experiment using PCR fragments. Four CpG-rich genomic loci (R1 R2, R3, and R4), and 2 CpG-free genomic loci (F1 and F2) were selected from the LC-Plasma genome. Three or four different fragments of each loci were selected and PCR primers were designed. The length and the copy number of CpG motifs in each fragments are shown in Additional file 1: Table S1. In total, 12 CpG-rich DNA fragments and 7 non-CpG fragments were amplified and subjected to pDCs stimulating assay. The IFN-α production of pDCs stimulated with these amplified fragments was shown in Fig. 1. Eleven of 12 CpG-rich DNA fragments strongly induced IFN-α production, while none of the CpG-free fragments induced IFN-α production. These results strongly suggested CpG motif is necessary for pDCs stimulation.

The CpG motif copy number is not strongly correlated to the pDCs stimulatory activity of DNA fragments from LC-Plasma.

Because the lengths of DNA fragments affect the transfection efficiency, we constructed another library of DNA fragments with uniform length from LC-Plasma. Fragments of approximately 200 bp with varied numbers of CpG motifs were randomly selected from the LC-Plasma genome (Additional file 2: Table S2). The pDCs stimulatory activity should be performed using DNA fragments with same length, The PCR-amplified fragments were subjected to assays for pDCs stimulatory activity.

**Fig. 2:**
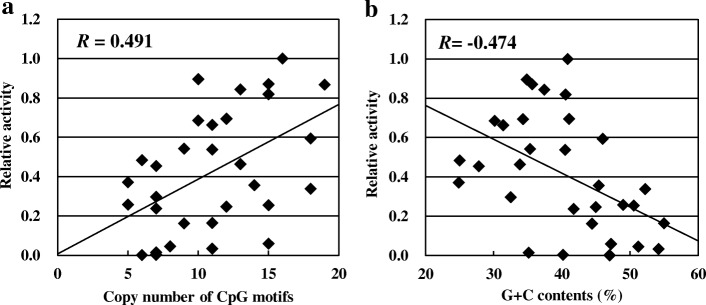
Correlation between immunostimulatory activity and the numbers of CpG motifs or G + C content contained in DNA fragments. Each dot depicts an independent 200 bp DNA fragment amplified from the LC-Plasma genome. Horizontal axes indicate **a**) the number of CpG motifs or **b**) G + C content contained in each DNA fragment. Vertical axes indicate the relative amount of IFN-α produced by BM-DCs stimulated by each type of DNA fragment

We analyzed the correlation between pDCs stimulatory activity and CpG motif copy number in each DNA fragment (Fig. 2a). The results showed that CpG motif copy number in the fragments was positively significantly correlated with activity (*p* < 0.01), and the correlation coefficient was *R* = 0.491, “moderate coefficient” defined by Guilford et.al. However, determination coefficient (*R*^*2*^*)* was only 0.24 which means another factor affects the pDCs stimulatory activity.

G + C content of DNA fragments from LC-Plasma is negatively correlated with pDCs stimulatory activity.

**Fig. 3:**
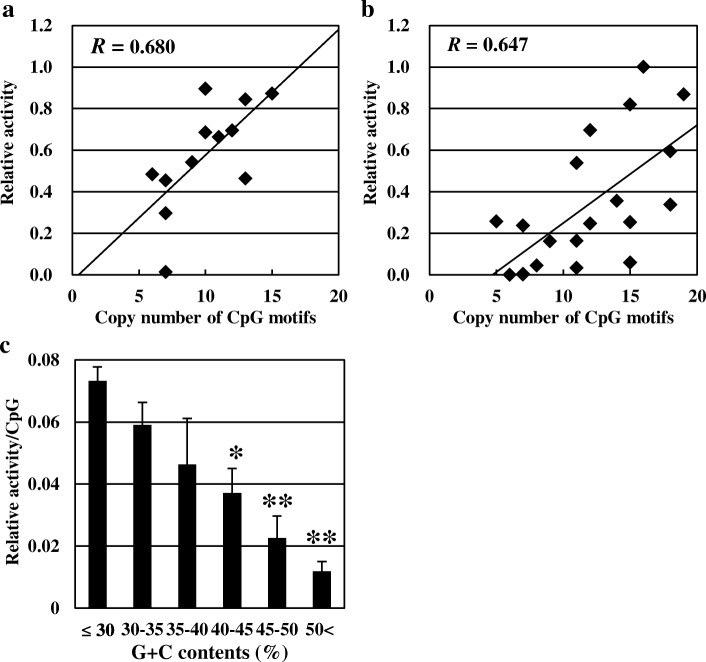
Correlation between the copy numbers of CpG motifs and immunostimulatory activity in 200 bp DNA fragments stratified by G + C contents. Fragments in Fig. 2 are stratified by G + C contents. **a** Each dot depicts an independent 200 bp amplified from the LC-Plasma genomic regions with G + C < 40% shown in Fig. 2. **b** Each dot depicts an independent 200 bp amplified from the LC-Plasma genomic regions with G + C ≥ 40%. **c** 200 bp DNA fragments amplified from the LC-Plasma genomic regions were stratified into 5 groups based on the copy numbers of CpG motifs and their pDCs stimulatory activities were compared. Bar depicts the standard deviation (S.D.). Bars with different notation exhibit significant differences (* *p* < 0.05, ** *p* < 0.01)

We then studied the relation of the G + C contents of DNA fragments with the level of pDCs stimulatory activity. A significant negative correlation between pDCs stimulatory activity and G + C contents of the fragment (*R* = − 0.474, *p* < 0.01, Fig. 2b) was observed. We performed bilayer stratified analysis based on G + C contents and compared the relation between the copy number CpG motifs and pDCs stimulatory activity. The DNA fragments into the low-G + C group composed of fragments with G + C < 40%, and the high-G + C group composed of fragments with G + C ≥ 40%. (Fig. 3a and b). The correlation coefficient was increased in both of the low-G + C group (*R* = 0.680, *p* < 0.01) and the high-G + C group (*R* = 0.647, *p* < 0.01). The degree of pDCs stimulatory activity per copy of CpG motifs was higher in the low-G + C group.

We also stratified DNA fragments into groups based on G + C contents as follows: < 30%, ≥30 to < 35%, ≥35 to < 40%, ≥40 to < 45%, ≥45 to < 50% and ≥ 50%. Stepwise reduction in pDCs stimulatory activity was observed, with a stepwise increase in G + C contents (Fig. 3c). We performed one-way ANOVA and Dunnet test. The results revealed that the levels of pDCs activity resulting fromv stimulation by fragments with G + C contents of ≥40 to < 45%, ≥45 to < 50%, and ≥ 50% were significantly lower compared to the activity induced by fragments with < 30% G + C. We also performed correlation analyses using randomly synthesized 300 bp fragments. Similar results were observed again (Additional file 3: Figure S1). These results strongly suggested that G + C content of DNA fragment is another essential factor to affect high level of pDCs stimulatory activity.

Total copy number of CpG motifs in the genome DNA is not strongly correlated to the pDCs stimulatory activity of LC-Plasma.

We carried out in silico analysis to investigate the relation between the copy number of CpG motifs and pDCs stimulatory activity. The total copy number of CpG motifs in the genome of *L. lactis* LC-Plasma was measured and compared to those of in the genomes of *Lactobacillus rhamnosus* ATCC 53103, and *Bifidobacterium longum* NCC 2705 which showed low pDCs stimulatory activity in a previous study [5]. The results suggested that the number of CpG motifs in the LC- Plasma is three times smaller than that in the ATCC 53103 and four times smaller than that in NCC2705 (Table 1). We also measured the three of the pDCs-activating motifs, and two of B cell activating motifs in the genome of these LAB (Table 1). The results showed that the genome of ATCC 53103 contained 3.7 to 5.7 fold greater copy number of pDCs activating motifs and 1.7 to 5.7 fold greater copy number of B cells activating motifs than that of the genome of LC-Plasma. The genome of NCC2705 contained 5.6 to 17.4 fold greater copy number of pDCs activating motifs and 1.5 to 2.8 fold greater copy number of B cells activating motifs than that of the genome of LC-Plasma. These results suggested the copy number of CpG motifs is not strongly related to the level of pDCs stimulatory activity of LC-Plasma.

**Table 1:** CpG motifs and copy numbers in the whole genome of LAB strains. Copy number of typical CpG motifs in the whole genome of *L. lactis* LC-Plasma, *L. rhamnosus* ATCC 53103, and NCC2706 were determined as described in Methods

			Bacterial strains		
			*Lactococcus lactis* LC-Plasma	*Lactobacillus rhamnosus* ATCC 53103	*Bifidobacterium longum* NCC2705
pDC activating		RRCGRYCGYY	51	195	288
		RYCGYRTCGYR	8	54	139
		RYCGRY	3072	17,681	25,057
B cell activating	optimal for mice	RRCGYY	4691	13,169	13,118
	optimal for human	GTCGTT	882	1534	1295
	CpG hexamer	NNCGNN	61,462	180,144	240,176

Comparing the pDCs stimulatory activity of single-stranded DNA.

**Fig. 4:**
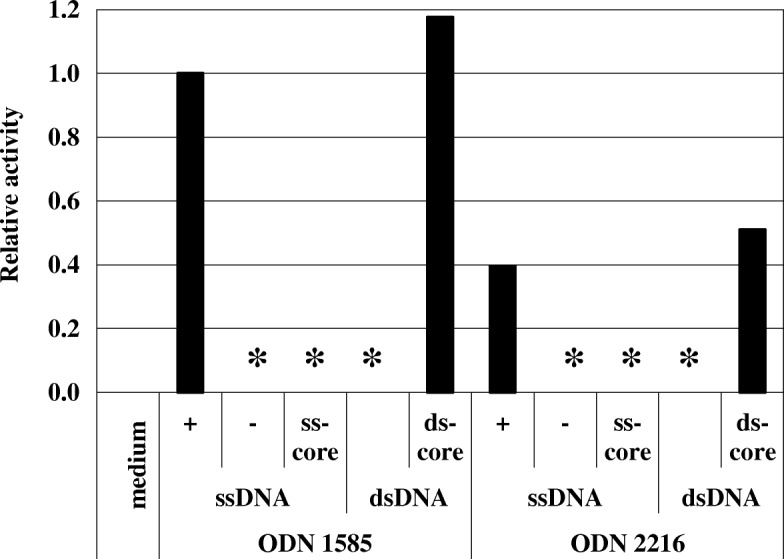
IFN-α production induced by single- or double-stranded forms of synthetic oligonucleotides. Single-stranded DNA oligomers (ssDNA) with phosphodiester bonds were synthesized, based on sequences of ODN1585 and ODN2216 (Invivogen, San Diego, CA, USA). Double-stranded DNA oligomers (dsDNA) were prepared by annealing sense and antisense strands of ssDNA. Each synthesized oligomer (2 μg) was tested on Flt3L induced BM-DCs, and the production of IFN-α was measured. +: sense strand; −: antisense strand; ss-core: 6-base CpG motif of sense strand; ds-core: Hybrid of sense strand DNA oligomer with antisense strand of 6 base CpG motif. *not detected

Because G + C content is directly related to the dissociation temperature of ds-DNA fragments, we evaluated pDCs stimulatory activity induced by synthetic oligonucleotides in single-stranded (ss) or double-stranded (ds) form. Two ss-CpG oligomers were synthesized, based on the sequences of ODN 1585 and ODN 2216 (InvivoGen, San Diego, CA, USA). As shown in Fig. 4, both oligonucleotides induced pDCs stimulatory activity, while their complementary sequences did not. We also synthesized the ds-form of ODN 1585 and ODN 2216, by annealing the normal and complementary strands. Interestingly, neither ODN 1585 nor ODN 2216 induced pDCs stimulatory activity in ds forms. In addition, the sense ODN hybridized with the antisense 6 bp sequence of the core CpG motif induced high pDCs stimulatory activity. These results suggest that an ss-CpG oligomer is more efficient at stimulating pDCs than a ds-CpG oligomer. The results also suggest that strong hybridization affinity between complementary strands might reduce the pDCs stimulatory activity of CpG motifs.

In silico analysis of the copy number of CpG motifs in whole genome and low-G + C region of the genome of LAB species.

**Fig. 5:**
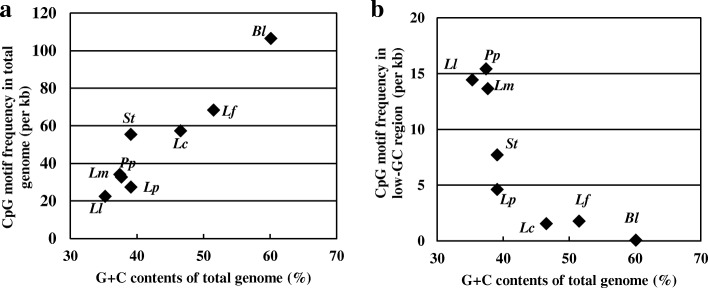
CpG motifs frequency in the genome of species of LAB. The frequency in each genome was depicted as a dot: **a**) whole genome and **b**) G + C < 40% . *Ll*; *Lactococcus lactis* LC-Plasma, *Pp*; *Pediococcus pentosaceus* ATCC 25745, *Lm*; *Leuconostoc mesenteroides* ATCC 8293, *St*; *Streptococcus thermophilus* CNRZ 1066, *Lp*; *Lactobacillus plantarum* WCFS1, *Lc*; *Lactobacillus casei* ATCC 334, *Lf*; *Lactobacillus fermentum* IFO 3956, *Bl*; *Bifidobacterium longum* NCC 2705

We investigated the frequency of CpG motifs in whole genomes and in the low-G + C region (< 40% of G + C contents) of the genome (Fig. 5a). A linear increase of frequency of CpG motifs was observed with increasing G + C content of whole genomes. On the contrary, the frequency of CpG motifs localized to low-G + C regions of the genome showed an inverse correlation with the G + C content of whole genomes (Fig. 5b). Three species (*Lactococcus lactis* subsp. *lactis*, *Pediococcus pentosaceus,* and *Leuconostoc mesenteroides*) with the genomes of low G + C contents (35.2 to 37.7%) contains 20 copies / kb CpG motifs in their low-G + C regions, while the other four species (*L. plantarum*, *L. casei*, *L. fermentum*, and *Bifidobacterium longum*) with the genomes of high G + C contents (46.6 to 60.1%) contains less than 10 copies/kb CpG motifs in their low-G + C regions.

The copy number of CpG motifs in the low-G + C region of the genome was closely related to the pDCs stimulatory activity of LAB cells.

As pDCs have been shown to recognize pathogens either by TLR9 or TLR7 and not by other TLRs [30], we hypothesized that the frequency of CpG motifs in the low-G + C regions affects the overall pDCs stimulatory activity of LAB. We investigated the differences of pDCs stimulatory activity between strains of LAB species using whole cells (Table 2). The wide variations of strains-based-activity were observed in each species. It was also observed that the frequencies of high-activity-strains were clearly different between the species. Two of 3 strains belonging to *L. lactis* subsp. *lactis* strains, two of 10 *L. mesenteroides* strains, and five of 19 *P. pentosaceus* strains induced marked (> 100 pg/mL) production of IFN-α. In silico analysis revealed that the genome of these three species have high CpG motif frequencies in the low-G + C regions (< 20 copy per kb). On the contrary, none of the LAB strains showing a lower frequency of CpG motifs in low-G + C regions (> 20 copy per kb), including *L. plantarum*, *L. casei*, and *L. fermentum*, exhibited significant stimulatory activity. The means of activity was also higher in the three strains compared to others. These results strongly suggest that the pDCs stimulatory activity of a bacterial strain depends on the copy number of CpG motifs in the low-G + C region of the genome and not on the copy number over the entire genome.

**Table 2:** pDCs stimulatory activity of LAB strains with varied GC contents in genome DNA

Species	Strains		IFN-α (pg/mL)*	CpG frequency (per kb)**	GC (%)	High***
*Lactococcus lactis *subsp. *lactis*	LC-Plasma		404.4	22.3	35.3	2/3
	JCM 20101		389.8			
	ATCC 15577		12.2			
		mean ± S.D.	271.0 ± 222.4			
*Pediococcus pentosaceus*	JCM 2026		84.0	28.4	37.4	5/19
	JCM 20109		353.3			
	JCM 20314		102.1			
	JCM 20459		5.5			
	NBRC 3182		59.1			
	NBRC 3891		15.8			
	NBRC 3892		116.4			
	NBRC 3893		32.2			
	NBRC 3894		46.0			
	NBRC 12229		18.7			
	NBRC 12230		7.9			
	NBRC 12232		14.8			
	NBRC 12318		59.8			
	NBRC 101982		404.4			
	NBRC 101983		5.6			
	NBRC 101984		5.9			
	NBRC 101985		5.8			
	NBRC 101986		172.3			
	NBRC 101987		34.3			
		mean ± S.D.	81.3 ± 114.5			
*Lenconostoc mesenteroides*	NBRC 3349		3.2	27.1	37.7	2/10
	NBRC 3426		57.3			
	NBRC 3832		353.5			
	NBRC 12060		4.2			
	NBRC 100495		5.7			
	NBRC 100496		8.1			
	NBRC 102497		36.0			
	NBRC 102480		74.2			
	NBRC 102481		167.0			
	NBRC 107766		4.4			
		mean ± S.D.	71.4 ± 111.6			
*Lactobacillus* low GC (< 40%)						
*Lactobacillus acidophilus*	JCM 1132		11.7	15.4	34.7	0/5
*Lactobacillus plantarum*	JCM 1551		16.7	4.6	39.1	
	JCM 6651		55.2			
	JCM 8341		4.0			
	JCM 20110		9.8			
		mean ± S.D.	19.5 ± 20.5			
*Lactobacillus* high GC (> 40%)						
*Lactobacillus casei*	ATCC 393		5.6	1.5	46.6	0/4
*Lactobacillus rhamnosus*	ATCC 53103		3.9	4.0	47.0	
*Lactobacillus fermentum*	NBRC 3959		4.4	1.9	51.5	
	NBRC 3961		5.2			
		mean ± S.D.	4.8 ± 0.8			

*IFN-α producing activity of whole cells of LAB strains

**CpG frequency in the low G + C region of the genomes. Strains used for calculation are shown in the Methods

***Number of high (> 100 pg/mL)-IFN-α-producing strains per tested strains

The origin and cultivation methods of the strains are shown in the Methods

We also carried out a statistical analysis of species-based pDCs stimulatory activity using Steel-Dwass method. Significant differences were observed between *L. lactis* to *P. damnosus*, *L. mensteroides*, and *Lactobacillus* low G + C species (*p* = 0.011, *p* = 0.033, *p* = 0.032, respectively). Marginally significant difference was also observed between *L. lactis* and *Lactobacillus* high-G + C species (*p* = 0.056). In addition, *P. pentosaceus* and *L. mesenteroides* also showed significant difference to *Lactobacillus* high-G + C species (*p* = 0.016, *p* = 0.033, respectively).

## Discussion

At the beginning of this study, we hypothesized that CpG copy number in the genome might correlate with pDCs stimulatory activity and that LC-Plasma may contain a greater copy number of CpG motifs and/or some special sequences containing CpG motifs. However, using DNA fragments and in silico analysis, our data did not support this hypothesis. In DNA fragment analysis, the CpG motifs proved to be necessary for pDCs stimulation. However, the correlation between the copy number of the CpG motifs and pDCs stimulatory activity was weak. For genome analysis, we could not find a greater copy number of total CpG motifs nor three of consensus sequences that have been reported as pDCs in the genome of LC-Plasma. During the process of DNA fragment analysis, we found that one fragment with very low pDCs stimulatory activity had high G + C content (Fig. 1, R4–2, G + C content = 50.7%). We hypothesized that the G + C content of DNA fragments might be critical for pDCs stimulatory activity and investigated the relationship between CpG copy number and G + C content on pDCs stimulatory activity using the same size of aligned DNA fragments. The results showed that G + C content had a negative correlation with pDCs stimulatory activity. The stratification of DNA fragments based on G + C content made the correlation between the copy number of CpG motifs and the pDCs stimulatory activity stronger.

Our results also showed that the CpG fragment lost its pDCs stimulating activity by annealing to the complementary whole strand, while annealing of the core sequence of the CpG motif did not reduce pDCs stimulating activity. This suggested that dissociation is important for the CpG-motif to stimulate pDCs. A recent study of the crystallised 3D structure of TLR9 suggested that single-stranded oligonucleotides bound to TLR9 could act as DNA agonists [31]. It is possible that the G + C content of DNA affects the dissociation of ds-DNA fragments and the consequent interaction with TLR9, which is then followed by activation of pDCs. However, some investigators insist that oligonucleotides cannot occur in single stranded forms [32], or that duplex structures are required for recognition by TLR9 [33, 34]. It has been suggested that the DNA sequence proximal to the CpG motifs is also important for activation, since we showed that the fragment did not lose its activity by annealing the complementary strand of the core CpG motif. Additional studies are needed to clarify whether single-stranded property is a key factor for pDCs activation.

We also investigated whether G + C content has an effect on the pDCs stimulatory activity of LAB cells. We found that LAB species with a higher frequency of CpG motifs in the low-G + C regions of the genome (> 20 copy per kb) were more likely to promote high pDCs stimulatory activity. Our previous report suggested that the CpG motifs are the most important MAMPs of LAB strains for pDCs stimulation [5]. The results in this study suggested that the copy number in the low-G + C regions strongly affects the pDCs stimulatory activity of whole cell LAB strains. In effect, we presented an in silico screening method of bacteria with high pDCs stimulatory activity at the species level. We showed that two new species, *Pediococcus pentosaceus* and *Leuconostoc mesenteroides,* both have higher pDCs stimulatory activity, which is true for *L. lactis* subsp. *lactis*. It should be noted that these two species are not genetically similar to *L. lactis* [35]. According to our stratified screening method, further screening may reveal other species with high pDCs stimulatory activity.

The effect of G + C content on immunostimulatory activity has not yet been fully studied for CpG motifs. Yamamoto et al. isolated DNA from bacteria, viruses, invertebrates, vertebrates, and plants. They investigated the natural killer (NK) stimulatory activity of DNA samples but no correlation was observed between G + C content and activity [36]. To the best of our knowledge, this is the first study to demonstrate that the G + C content of DNA fragments has a direct effect on the immunomodulatory activity of pDCs.

Our data also suggested that there are independent properties of LAB other than the copy number of CpG motifs in low-G + C region, which can contribute to immunostimulatory activity. The activity of *L. lactis* was significantly higher than that observed with *Pediococcus pentosaceus* and *Leuconostoc mesenteroides.* However, it should be noted that the copy number of CpG motifs in the low G + C region of the aforementioned species were higher than that of *L. lactis*. These results suggested that other factors might have affected the variation in pDCs stimulatory activity, such as the bacterial cell’s affinity to pDCs or a more suitable size of cell envelop for phagocytosis. These results may also have affected the variation in pDCs stimulatory activity at the strain level, since we observed a wide variety of activities in strains within single species.

Our findings may be generally applicable for bacteria other than LABs. Kant et al. performed a bioinformatics study of gut bacteria genomes. They suggested that the number of CpG motifs were strongly correlated with G + C content in a negative fashion, which was also observed in this study. Analysis of CpG motifs in the low-G + C region of gut bacteria genomes may help to understand the effect of each bacterial type on pDCs in the gut [35]. Ménard et al. showed that CpG-rich DNA fragments with high G + C content from *Bifidobacterium longum* were effective for macrophage activation [36]. However, when we tested CpG-rich DNA fragments with high G + C content from *B. longum* on BM-derived DCs, we did not found high activity (data not shown). It would also be a great interest to study the effect of G + C contents of the genome on other immunocytes in future.

## Conclusions

In this study, we demonstrated for the first time that there was a strong correlation between the CpG copy numbers in the low-G + C region of DNA fragments from bacterial genomes and pDCs stimulatory activities of the fragments. Our study provides a new perspective on the structure of DNA fragments that are able to activate pDCs via the TLR9-Myd88 pathway. The information from this study should be useful for designing new DNA fragments, including phosphodiesterbond-DNA oligomers containing CpG motifs and DNA-containing vaccines. This work also detailed an in silico screening method for identifying bacterial species that are able to activate pDCs. Additional investigations and applications of our hypothesis may lead to a more detailed understanding of host-bacterium interactions via TLR9 for other bacteria, immune reactions, and immunocytes.

## Methods

### Bacterial strains

The bacterial strains used in this study, *Lactococcus lactis* LC-Plasma and *Lactobacillus rhamnosus* ATCC 53103, were purchased from the collections held at the Japan Collection of Microorganisms (JCM) and American Type Culture Collection (ATCC), respectively. Other bacterial strains used in the screening assay were purchased from JCM, ATCC, or NITE Biological Resource Center (NBRC).

Cultures of bacterial strains were grown at 30 °C or 37 °C for 48 h in De Man, Rogosa, and Sharpe (MRS) medium (BD Biosciences) or GAM medium (Nissui), which were prepared according to the suppliers’ instructions.

### Preparation of DNA fragments

Genomic DNAs were extracted and purified from bacterial cultures, using QIAGEN Genomic-tip 500/G (Qiagen) according to manufacturer’s instruction. The purity of DNA was confirmed using Nano drop (Thermo Fisher Scientific). PCR amplifications of selected sequences, which were based on the results of our in silico analysis, were performed using the GeneAmp PCR System (Applied Biosystems), with primers designed according to the *L. lactis* LC-Plasma genome sequence. PCR was performed using TaKaRa Ex *Taq*® (TaKaRa), according to the manufacturer’s instructions, using 10 ng of DNA template in 50 μl of reaction mixture containing primers at a concentration of 0.5 μM. The following thermal cycling profile was used: 5 min at 94 °C followed by 35 cycles of 30 s at 94 °C for denaturation, 30 s at hybridization temperatures based on the primers, and 30 s at 72 °C for extension; and then a final 7-min extension phase at 72 °C.

The PCR products were purified using QIAquick PCR Purification Kit (Qiagen), according to the manufacturer’s instructions, using 50 μl of elution solution. Each eluent was evaporated and concentrated on a DNA SpeedVac (Thermo Scientific). The concentrated DNA solutions were assessed by NanoDrop 2000 (Thermo Scientific), and the DNA concentration was adjusted to 10 mg/mL using double-distilled water.

The oligonucleotide sequences used for amplification; and the length, G + C content, and number of CpG motifs contained in the amplicon are shown in Additional file 1: Table S1. The draft genome sequence of *L. lactis* LC-Plasma was available to the public [37] and was used for the design of primers and other purposes.

### Bone marrow (BM)-derived DC cultures

Four to 8-week-old female BALB/c wild-type mice were purchased from CLEA Japan. Flt3L-induced DCs were generated as follows. The mice were sacrificed using 5.0% isoflurane delivered with a precision vaporizer followed with cervical dissociation by a well-trained operator. BM cells were extracted from BALB/c mice, and erythrocytes were removed by brief exposure to 0.168 M NH_4_Cl. Cells were cultured at a density of 5 × 10^5^ cells/mL for 7 days in RPMI 1640 medium (Life Technologies) containing 1 mM sodium pyruvate (Life Technologies), 2.5 mM HEPES (Life Technologies), 100 U/mL penicillin/100 μg/mL streptomycin (Life Technologies), 50 μM 2-ME (Life Technologies), 10% fetal calf serum (Life Technologies), and 100 ng/mL Flt3L (R&D Systems).

### Stimulating assay for pDCs

BM-derived DC cultures were stimulated with purified PCR products at a final concentration of 2 μg/mL in the presence of FuGENE® HD Transfection Reagent (Promega) according to the manufacturer’s instructions. Briefly, FuGENE HD was added to the RPMI 1640 medium with 1000-fold dilution in final. Then, purified PCR products were added and the mixture was incubated for 5 min at room temperature. Each incubated mixture (50 μL) was added to 500 μL of culture medium containing BM-derived DCs at a density of 5.0 × 10^5^ cells/mL. After overnight incubation at 37 °C in an atmosphere containing 5% CO_2_ and 95% air, the cell cultures were collected and centrifuged to obtain culture supernatants. The supernatants were stored at − 80 °C until analysis. IFN-α concentration was measured using the VeriKine™ IFN-α ELISA Kit (PBL Assay Science), according to the manufacturer’s instructions. For the experiment using oligomers (less than 50 bp nucleotides) or whole cells, we did not use FuGENE® HD. LAB whole cells or oligomer (10 μg/ml and 2.0 μM, respectively) were added to the cultures of BM-derived DCs. Cultures were incubated for 48 h and the supernatants were submitted for IFN-α analysis as previously described [5].

### In silico analysis of bacterial genomes

In silico analysis was performed using Genetyx ver.9 software (GENETYX).

We searched for 5′-purine-purine-CG-pyrimidine-pyrimidine-3′ (5′-RRCGYY-3′) and 5′-purine-TCG-pyrimidine-pyrimidine-3′ (5′-RTCGYY-3′), and the. Total number of CpG hexamers (5′-NNCGNN-3′) in each genome was also calculated. Genomic regions with low G + C content (e.g. G + C < 40%) were extracted using the source code that we created, based on the Perl Programming Language. When any 200 bp fragment was calculated with a G + C content ≥40%, genomic regions containing that fragment were designated as high-G + C regions. The genome sequence data of *Lactococcus lactis* subsp. *lactis* LC-Plasma, *Leuconostoc mesenteroides* NBRC 100496 (synonym of ATCC 8293), *Lactobacillus acidophilus* NCFM, *Lactobacillus plantarum* WCFS1, *Lactobacillus casei* ATCC 334, *Lactobacillus fermentum* IFO 3956, *Lactobacillus rhamnosus* ATCC 53103, and *Pediococcus pentosaceus* ATCC 25745 were obtained from GENBANK and were used for in silico analysis.

## Additional files

Additional file 1: **Table S1.** Primer sequences used for the amplification of CpG-rich or CpG-free fragments. Primer sequences and the number of copies of CpG motifs in the amplicon used for verifying involvement of CpG motifs. (XLSX 10 kb)

Additional file 2: **Table S2.** 200 bp DNA fragments obtained from *L. lactis* LC-Plasma. (XLSX 13 kb)

Additional file 3: **Figure S1.** Correlation between the copy numbers of CpG motifs in 300 bp DNA fragments and IFN-α. Each dot depicts an independent 300 bp DNA fragment amplified from the LC-Plasma genome. Horizontal axes indicate the number of CpG motifs contained in each DNA fragment for A) all fragments, B) low-G + C fragments and C) high-G + C fragments. Vertical axes indicate the relative activity produced by BM-DCs stimulated by each DNA fragment (PPTX 100 kb)

## Abbreviations

ATCC: American type culture collection
BM: Bone marrow
DCs: Dendritic cells
ds: Double-stranded
Flt3L: Fms-like tyrosine kinase 3 ligand
IFN-α: Interferon-alpha
INH-ODN: Immuno-inhibitory oligonucleotide
ISS-ODN: Immunostimulatory oligonucleotide
JCM: Japan Collection of Microorganisms
LAB: Lactic acid bacteria
LC-Plasma: *Lactococcus lactis* strain Plasma
LPS: Lipopolysaccharides
LTA: Lipoteichoic acids
MAMPs: Microbe Associated Molecular Patterns
MRS: De Man, Rogosa, and Sharpe
MxA: Myxovirus resistance A
NBRC: NITE Biological Resource Center
NK: Natural killer
pDCs: Plasmacytoid dendritic cells
ss: Single-stranded
TLR9: Toll-like receptor 9
TLRs: Toll-like receptors
