# Supplementary figures and images for: Characterization of genomic DNA of lactic acid bacteria for activation of plasmacytoid dendritic cells
Source: BMC Microbiol. 2019 May 6;19:88. doi: 10.1186/s12866-019-1458-y (PMC6501324; doi:10.1186/s12866-019-1458-y)

## Slide 1
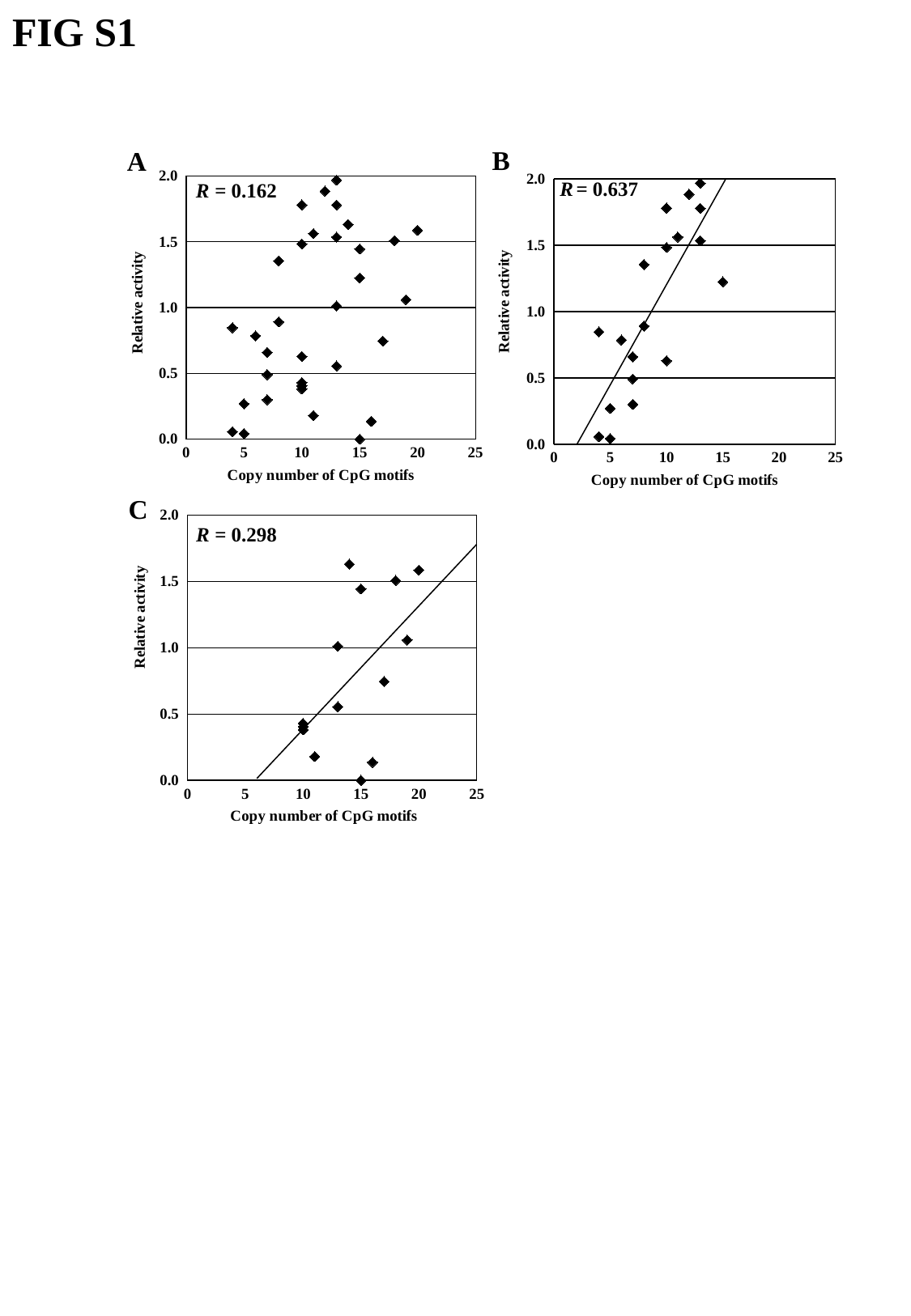

# FIG S1
B
A
### Chart
| Category | CpG |
|---|---|
### Chart
| Category | CpG |
|---|---|C
### Chart
| Category | CpG |
|---|---|

Supplement: Supplementary file 3 — Figure S1. Correlation between the copy numbers of CpG motifs in 300 bp DNA fragments and IFN-α. Each dot depicts an independent 300 bp DNA fragment amplified from the LC-Plasma genome. Horizontal axes indicate the number of CpG motifs contained in each DNA fragment for A) all fragments, B) low-G + C fragments and C) high-G + C fragments. Vertical axes indicate the relative activity produced by BM-DCs stimulated by each DNA fragment (PPTX 100 kb) [file 12866_2019_1458_MOESM3_ESM.pptx]
